# Latency duration of preterm premature rupture of membranes and neonatal outcome: a retrospective single-center experience

**DOI:** 10.1007/s00431-021-04245-2

**Published:** 2021-10-04

**Authors:** Hanna Müller, Ann-Christin Stähling, Nora Bruns, Christel Weiss, Maria Ai, Angela Köninger, Ursula Felderhoff-Müser

**Affiliations:** 1 Neonatology and Pediatric Intensive Care, Department of Pediatrics, University of Marburg, Baldingerstraße, 35043 Marburg, Germany; 2 Department of Pediatrics I, Neonatology, Pediatric Intensive Care, Pediatric Neurology, University Hospital Essen, University Duisburg-Essen, Hufelandstr. 55, 45147 Essen, Germany; 3 Clinic for Urology and Pediatric Urology, Marien-Hospital Marl, KKRN GmbH, Hervester Str.57, 45768 Marl, Germany; 4 Department of Medical Statistics and Biomathematics, University Hospital Mannheim, Theodor-Kutzer-Ufer 1-3, 68167 Mannheim, Germany; 5 Department of Pediatrics, University Hospital of Erlangen, University of Erlangen-Nürnberg, Loschgestr. 15, 91054 Erlangen, Germany; 6 Department of Gynecology and Obstetrics, University Hospital Essen, University Duisburg-Essen, Hufelandstr. 55, 45147 Essen, Germany

**Keywords:** Chorioamnionitis, Neurological development, Preterm infant, Preterm premature rupture of membranes, Respiratory distress syndrome

## Abstract

In preterm premature rupture of membranes (PPROM), a decision between early delivery with prematurity complications and pregnancy prolongation bearing the risk of chorioamnionitis has to be made. To define disadvantages of delayed prolongation, latency duration of PPROM in expectantly managed pregnancies was investigated. We included those PPROMs > 48 h leading to preterm birth prior 37 weeks’ gestation and retrospectively analyzed 84 preterm infants fulfilling these criteria. The association between latency duration/appearance of PPROM and respiratory outcome (primary outcomes) and neurological outcome (secondary outcomes) was investigated. The study showed that latency duration of PPROM is not associated with clinical or histological chorioamnionitis (*p* = 0.275; *p* = 0.332). As the numerous clinical parameters show multicollinearity between each other, we performed a multiple regression analysis to consider this fact. Respiratory distress syndrome is significantly associated with gestational age at PPROM (*p* < 0.001), and surfactant application is significantly associated with PPROM duration (*p* = 0.014). The other respiratory parameters including steroids and diuretics therapy, bronchopulmonary dysplasia, and the neurological parameters (intraventricular hemorrhage, Bayley II testing at a corrected age of 24 months) were not significantly associated with PPROM duration or gestational age at PPROM diagnosis.

*Conclusion*: Latency duration of PPROM was not associated with adverse neonatal outcome in expectantly and carefully managed pregnancies, but respiratory distress syndrome was pronounced. The observed effect of pronounced respiratory distress syndrome can be treated with surfactant preparations and was not followed by increased rate of bronchopulmonary dysplasia.

**Table:** 

**What is Known:** *• In case of preterm premature rupture of membranes, a decision between pregnancy prolongation with the risk of chorioamnionitis and early delivery with prematurity complications has to be made.* *• Chorioamnionitis is a dangerous situation for the pregnant woman and the fetus.* *• Impaired neurodevelopmental outcome is strongly correlated with pronounced prematurity due to the increased rate of serious complications.*	
**What is New:** *• Respiratory distress syndrome is significantly associated with gestational age at PPROM, and surfactant application is significantly associated with PPROM duration.* *• Latency duration of PPROM is not associated with adverse respiratory neonatal outcome (therapy with continuous positive airway pressure, therapy with diuretics and/or steroids, bronchopulmonary dysplasia) in expectantly and carefully managed pregnancies.* *• Intraventricular hemorrhage and Bayley II testing at a corrected age of 24 months are not associated with latency duration of PPROM when pregnancies are carefully observed.*

**Supplementary information:**

The online version contains supplementary material available at 10.1007/s00431-021-04245-2.

## Introduction

Preterm premature rupture of membranes (PPROM), defined as premature rupture of membranes occurring before 37 weeks of gestation, is a serious complication during pregnancy. PPROM enables ascending infections from the vagina of pregnant women and conceivably leads to chorioamnionitis, a dangerous situation for mother and fetus. Intra-amniotic inflammation is observed in approximately 40% of women with PPROM [1]. Furthermore, PPROM together with preterm labor is frequently the consequence of sub-clinical chorioamnionitis [2, 3]. Severe chorioamnionitis is associated with an increased short- and long-term morbidity and mortality of the affected newborns [3, 4]. Neonatal morbidity includes pulmonary complications (respiratory distress syndrome, bronchopulmonary dysplasia), fetal and neonatal brain injury (altered brain development, tissue loss, intraventricular hemorrhage, cystic periventricular leukomalacia, white matter damage), and adverse neurological development [1, 3, 5–8]. Rapid application of antibiotics to the women suffering from PPROM is initiated to prolong gestation and to reduce morbidity of the fetus/neonate [3, 9]. The minimal goal after diagnosis of PPROM includes prolongation of pregnancy for about 48 h to enable fetal lung maturation. Maternal administration of antenatal steroids could be followed by prolongation of gestation over a long time, thereby reducing prematurity and its associated complications. This balance between prevention of prematurity and avoidance of chorioamnionitis is limited, which makes timing of delivery very difficult [3, 10]. A recently formulated recommendation includes careful monitoring of women with PPROM before 37 weeks’ gestation even without contraindications, to prolong pregnancy and achieve better outcome [11]. The aim of this study was to investigate the effect of latency duration of PPROM on adverse respiratory and neurological outcomes in expectantly and carefully observed and treated pregnancies of preterm infants born prior 37  weeks’ gestation.

## Methods

### Patients

**Fig. 1:**
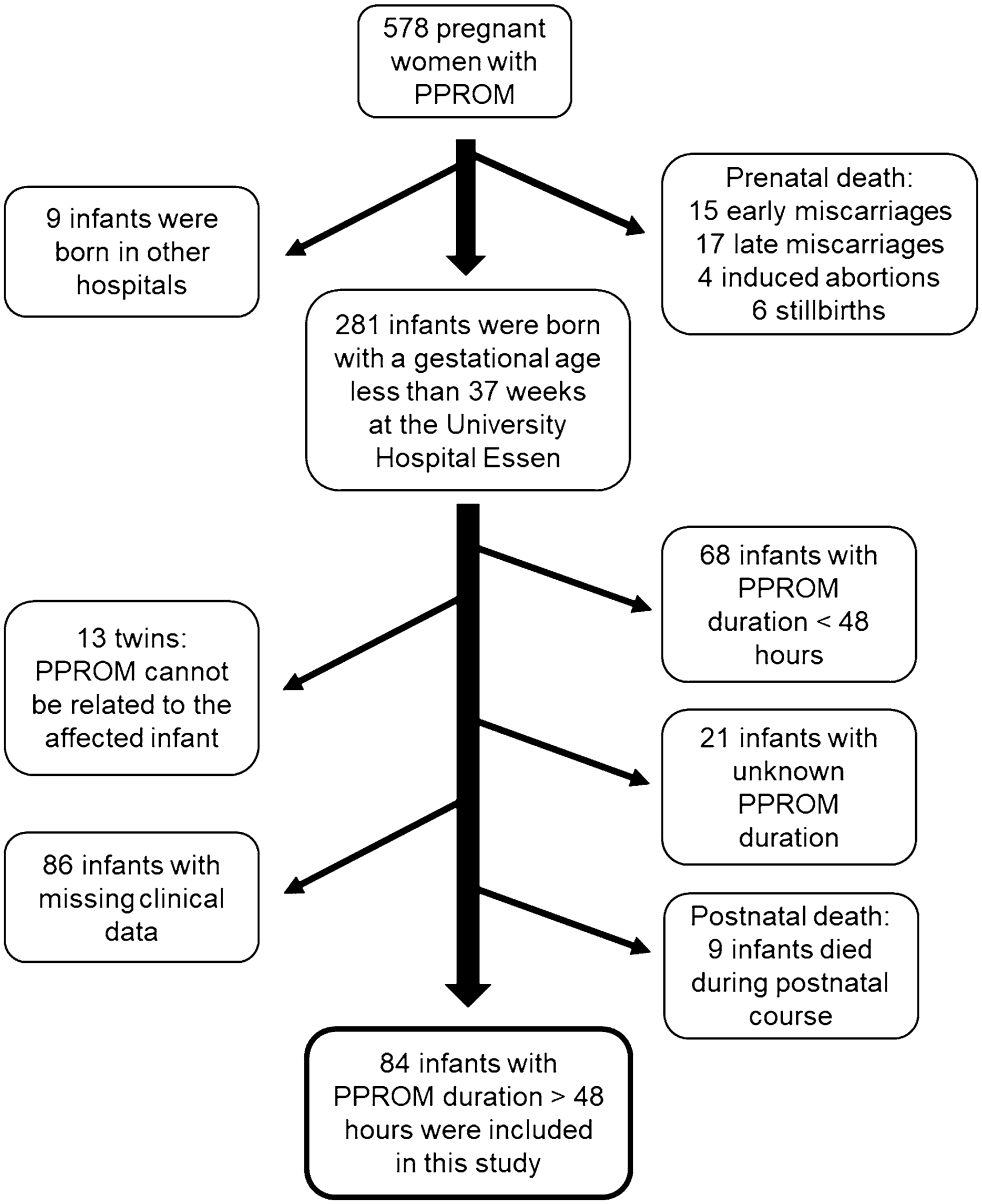
Flow chart of patient recruitment

We retrospectively analyzed 84 preterm infants born after PPROM and delivered between 2005 and 2014 at the University Hospital Essen. We included those PPROMs leading to preterm birth prior 37 weeks’ gestation and Fig. 1 illustrates patient recruitment. Clinical parameters of the infants were collected by evaluation of medical records. The study was approved by the local ethics committee in accordance with the Helsinki declaration (1964) and its later amendments (15-6521-BO). The patient anonymity was preserved. Parental consent was not necessary due to the retrospective character of this study and due to the fact that only the records of infants were used for analysis.

Gestational age at birth was defined as time elapsed between the first day of the last menstrual period and the day of delivery and was confirmed by ultrasound. The clinical definition of chorioamnionitis included maternal fever (temperature ≥ 38.0 °C) with at least one of the following clinical signs: abdominal tenderness, fetal tachycardia (continuous heart rate above 160/minute), maternal biochemical evidence of chorioamnionitis (increased maternal C-reactive protein (CrP) of at least 2.0 mg/dl, and/or leukocytosis (values above 15,000/μl)) or purulent vaginal discharge [3, 12]. Furthermore, histological examination of the placenta was performed in 58 patients. Mothers with PPROM received antibiotics according to a standard containing piperacillin and metronidazole or clindamycin in cases of an allergy against penicillin [3]. All patients received a microbiological swab of the cervix. If the detected bacteria showed resistance against the named antibiotics, substances were changed according to the antibiogram-resistogram. In cases with slightly increasing inflammation parameters in the blood, antibiotics were once changed towards meropenem with the aim of a prolongation of the pregnancy. The rationale to use meropenem was the broad range of efficacy against gram-positive and gram-negative as well as anaerobic bacteria and the very well tolerance by the patients. However, the final decision whether to wait or to initiate delivery or to perform a cesarean section was performed by a senior obstetrician. In cases of uterine contractions, patients received tocolytics (ß-mimetics like fenoterol intravenously 1.0–4.0 µg/min or calcium channel blockers like oral intake of nifedipine 40–160 mg/day).

We defined parameter of the respiratory outcome as primary outcomes, and we decided to evaluate several binary factors: respiratory distress syndrome, surfactant application, bronchopulmonary dysplasia, therapy with continuous positive airway pressure (CPAP), and therapy with diuretics and steroids. As secondary outcomes, we regarded the neurological parameters intraventricular hemorrhage and Bayley II testing at a corrected age of 24 months.

Respiratory distress syndrome was defined by X-ray in any child with respiratory insufficiency according to Giedion et al. [13]. We also focused on a potential association between bronchopulmonary dysplasia and duration of PPROM, because bronchopulmonary dysplasia is a serious and frequent complication of severe respiratory distress syndrome and of prolonged mechanical ventilation in very immature preterm infants. Two different bronchopulmonary dysplasia definitions were used. One bronchopulmonary dysplasia definition includes oxygen supplementation at day 28 of life, and the second and preferentially used bronchopulmonary dysplasia definition is characterized by oxygen need or respiratory support at an age of 36 postmenstrual weeks. Neurodevelopmental outcome (Mental and Psychomotor Developmental Indexes (MDI and PDI)) was determined in all infants using Bayley Scales of Infant Development, 2nd Edition (BSID II) at a corrected age of 24 months. In the case of MDI or PDI scores < 50, we used the value 45 to enable statistical analysis.

### Methods

The latency duration as well as appearance (gestational week) of PPROM were correlated to different clinical parameters of primary and secondary outcomes and in particular to the frequency of chorioamnionitis.

### Statistics

Statistical analysis was performed with SAS software, release 9.4 (SAS Institute Inc., Cary, NC, USA). Quantitative variables are presented by mean value and standard deviation together with median, minimum, and maximum. For qualitative factors, absolute and relative frequencies are given. Mean values of two independent groups have been compared with a two sample *t* test if data are approximately normally distributed. For skewed distributions Mann-Whitney *U* test has been used instead. Ordinally scaled parameters (i.e., maximal grade of respiratory distress) have been compared with Cochran-Armitage trend test. In order to compare relative frequencies, chi^2^ test or Fisher’s exact test has been performed. In order to quantify the strength of correlation of two quantitative variables, correlation coefficients according to Pearson were assessed. If one or both variables were ordinally scaled, coefficients according to Spearman were calculated instead. Furthermore, multiple logistic regression analysis was performed for the binary outcomes: respiratory distress syndrome, surfactant application, bronchopulmonary dysplasia, therapy with continuous positive airway pressure, therapy with diuretics, and therapy with steroids in order to investigate the influence of several parameters on the outcome simultaneously. For the multiple models, all variables have been considered which had been proven to be significant at a level of *α* = 0.10 in the univariable analyses. For the final model, variables had been selected using the “selection = stepwise” option. The goodness of these models has been quantified by the AUC (area under the curve) value. In general, test results with *p* values less than 0.05 have been regarded as statistically significant.

## Results

Tables 1 and 2 demonstrate demographic and clinical characteristics of patients and their mothers included in the study. The median of PPROM duration was 1 week, and PPROM was diagnosed between 14 and 34 weeks of gestation (Table 1). Median maternal C-reactive protein was only 1.05 mg/dl, and median C-reactive protein of the infants within the first 72 h of life was unremarkable with slightly higher interleukin 6 levels demonstrating an accurate clinical monitoring of pregnancy and a prompt initiation of birth in case of beginning chorioamnionitis.

**Table 1:** Demographic and clinical characteristics of the enrolled patients and their mothers

Variable	*n*	Mean ± SD	Median	Min	Max
Gestational age at birth (weeks)	84	29.7 ± 2.9	30.1	22.6	36.1
Birth weight (g)	84	1442 ± 546	1480	470	2940
Duration of PPROM (hours)	84	399.3 ± 545.8	168.0	48.0	2700.0
Duration of PPROM (weeks)	84	2.4 ± 3.2	1.0	0.3	16.1
Occurrence of PPROM (gestational age; weeks)	84	27.2 ± 4.4	28.0	14.4	34.9
Maternal CrP (mg/dl)	82	1.69 ± 2.12	1.05	0	9.6
Max. CrP of infants in the first 72 h of life (mg/dl)	76	0.3 ± 0.8	0	0	3.3
IL6 of infants in the first 72 h of life (pg/ml)	80	1000.4 ± 7929.3	3.7	0	70,925.0

*CrP* C-reactive protein, *IL6* interleukin 6, max maximum, min minimum, *PPROM* preterm premature rupture of membranes, *SD* standard deviation

**Table 2:** Antenatal steroid application, respiratory distress syndrome and surfactant application, frequency and therapy of bronchopulmonary dysplasia and intraventricular hemorrhage in the study population

Antenatal steroid application (data available in 83 mothers)	Cycles [*n*]	Mothers [*n* (%)]
	0	6 (7.2)
	1	51 (61.4)
	2	25 (30.1)
	3	1 (1.2)
Respiratory distress syndrome (data available in 80 infants) Median grade: 1	Max grade	Infants [*n* (%)]
	0	24 (30.0)
	1	24 (30.0)
	2	19 (23.8)
	3	10 (12.5)
	4	3 (3.7)
Surfactant application (data available in 84 infants)	Number of applications	Infants [*n* (%)]
	0	49 (58.3)
	1	18 (21.4)
	2	10 (11.9)
	3	4 (4.8)
	4	2 (2.4)
	5	1 (1.2)
Bronchopulmonary dysplasia (definition: day 28 of life, data available in 71 infants)	No [*n* %]	Yes [*n* %]
	58 (81.7)	13 (18.3)
Bronchopulmonary dysplasia (definition: 36 weeks PMA, data available in 78 infants)	No [*n *%]	Yes [*n* %]
	69 (88.5)	9 (11.5)
Therapy with diuretics (data available in 80 infants)	No [*n* %]	Yes [*n* %]
	72 (90.0)	8 (10.0)
Therapy with steroids (data available in 78 infants)	No [*n* %]	Yes [*n* %]
	71 (91.0)	7 (9.0)
Intraventricular hemorrhage (data available in 82 infants)	Grade	Infants [*n* (%)]
	0	74 (90.2)
	1	6 (7.3)
	2	1 (1.2)
	3	1 (1.2)

*Max* maximal, *PMA* postmenstrual age

### Latency duration of PPROM and chorioamnionitis

Histological evaluation of the placenta was performed in 58 out of 84 women (69%). In 2009, pathologic examination of placentas in the case of suspected chorioamnionitis was introduced leading to increased frequency of appropriate placental pathological examinations. Histological chorioamnionitis was diagnosed in 13 out of 58 examined placentas (supplemental Table 1). The duration of PPROM in the group with histological chorioamnionitis was 2.2 ± 3.8 weeks (mean ± SD; median 0.9 weeks; range 0.3–13.6 weeks) and in the group without histological chorioamnionitis 2.8 ± 3.5 weeks (mean ± SD; median 1.1 weeks; range 0.3–16.1 weeks) demonstrating no significant difference (*p* = 0.332; Mann-Whitney *U* test). Additionally, we examined the presence of clinically defined chorioamnionitis (supplemental Table 1). The duration of PPROM in the group with clinical chorioamnionitis (41 infants) was 2.6 ± 3.2 weeks (mean ± SD; median 1.0 weeks; range 0.3–13.9 weeks) and in the group without clinical chorioamnionitis (43 infants) 2.2 ± 3.3 weeks (mean ± SD; median 1.0 weeks; range 0.3–16.1 weeks) illustrating no significant difference (*p* = 0.275; Mann-Whitney *U* test).

Additionally, patients were divided into two groups (PPROM < 1 week or PPROM ≥ 1 week; Table 3) because this is a time with high discussion about prolongation in many hospitals as fetal lung maturation is finished and a good argumentation is necessary to reason further hospital stay of the pregnant woman. Univariable analyses revealed that these two groups differ significantly regarding gestational age of occurrence of PPROM (*p* < 0.001), maximal grade of respiratory distress syndrome (*p* = 0.010), and number of surfactant applications (*p* = 0.001). Multiple logistic regression analysis showed that only gestational age of occurrence of PPROM is significant. However, when using a multiple regression analysis with the quantitative outcome “duration of PPROM” besides the most important parameter gestational age at PPROM (*p* < 0.001), maximal grade of respiratory distress syndrome (*p* = 0.047) as well as number of surfactant applications (*p* = 0.049) revealed to be significant.

**Table 3:** Comparison of different clinical parameters due to PPROM duration < 7 days and ≥ 7 days

Variable	PPROM duration < 7 days (*n* = 41)	PPROM duration ≥ 7 days (*n* = 43)	*p* value
Birth weight (mean ± SD; range)^a^	1519 ± 552 (470–2300)	1368 ± 536 (560–2940)	0.206
Gestational age at birth (mean ± SD; range)^a^	29.9 ± 3.1 (22.6–33.9)	29.4 ± 2.7 (24.4–36.1)	0.441
Occurrence of PPROM (gestational age) (mean ± SD; range)^a^	29.2 ± 3.3 (21.9–33.3)	25.3 ± 4.6 (14.4–34.9)	< 0.001
Clinical chorioamnionitis [*n*; %]^b^	20/41 (49%)	21/43 (49%)	0.996
Histologic chorioamnionitis [*n*; %]^b^	7/25 (28%)	6/33 (18%)	0.375
Max. CrP of infants in the first 72 postnatal hours (median; range)^c^	0 (0–3.3)	0 (0.0–3.0)	0.288
Max. IL6 of infants in the first 72 postnatal hours (median; range)^c^	0 (0–1187)	8.6 (0–70,925)	0.354
Respiratory distress syndrome (no versus yes) [*n*; %]^b^	23/37 (62%)	33/43 (77%)	0.156
Max. grade of respiratory distress syndrome of the infants with respiratory distress syndrome (median; range)^d^	1 (0–4)	2 (0–4)	0.010
Intraventricular hemorrhage (grade) (median; range)^d^	0 (0–1)	0 (0–3)	0.162
Surfactant application (no versus yes) [*n*; %]^b^	11/41 (27%)	24/43 (56%)	0.007
Number of surfactant applications (median; range)^d^	0 (0–1)	1 (0–5)	0.001
Bronchopulmonary dysplasia (definition: 36 weeks PMA) [*n*; %] ^e^	5/40 (12.5%)	4/38 (11%)	1.000
Therapy with continuous positive airway pressure [*n*; %]^e^	2/39 (5%)	2/38 (5%)	1.000
Therapy with diuretics [*n*; %]^e^	5/41 (12%)	3/39 (8%)	0.713
Therapy with steroids [*n*]^e^	3/41 (7%)	4/37 (11%)	0.702
MDI score (mean ± SD; range)^a^	90.3 ± 22.1 (45–122)	100.4 ± 11.4 (78–113)	0.117
PDI score (mean ± SD; range)^a^	81.4 ± 17.1 (45–103)	92.3 ± 13.0 (69–111)	0.087

*CrP* C-reactive protein, *IL6* Interleukin 6, *Max* maximum, *MDI* mental development index, *PDI* psychomotor developmental index, *PMA* postmenstrual age, *PPROM* preterm premature rupture of membranes

^a^*t* test

^b^Chi^2^ test

^c^Mann-Whitney *U* test

^d^Cochran-Armitage trend test

^e^Fisher’s test

### Detection of bacteria in patients with early-onset sepsis and their mothers

Early-onset sepsis was diagnosed in 10 infants. Table 4 demonstrates the detection of bacteria in these infants and vaginal colonization of their mothers. In four of 10 infants with early-onset sepsis, a detection of bacteria was possible: *Escherichia coli* (three infants) and *Klebsiella pneumonia* (one infant). In three of the 10 corresponding mothers bacteria and/or candida were detected; the vaginal colonization of one mother included *E. coli* and *Candida glabrata*. In only two of these 10 infants (20%), bacteria detected in neonatal infection corresponded to the vaginal colonization of the mothers.

**Table 4:** Detection of bacteria in patients with early-onset sepsis and vaginal colonization of their mothers

No.	Chorioamnionitis		Max CrP	Max IL6	Maternal vaginal colonization (swabs)	Neonatal detection of bacteria
	Histological	Clinical	During the first 72 h			
1	No exam	Yes	2.3	317	n a	No bacteria
2	No	Yes	1.0	477	n a	*Klebsiella pneumoniae*
3	Yes	Yes	2.3	70,925	*E. coli*	*E. coli*
4	No	Yes	2.5	905	No bacteria/candida	*E. coli*
5	No	Yes	3.0	3685	n a	n a
6	Yes	Yes	1.5	n.a	*Candida albicans*	No bacteria/candida
7	No	Yes	3.3	1187	n a	no bacteria
8	No exam	Yes	2.4	301	*E. coli*, *Candida glabrata*	*E. coli*
9	No	Yes	1.5	10	No bacteria	No bacteria
10	No	Yes	1.3	30	No bacteria	No bacteria

*CrP* C reactive protein, *IL6* Interleukin 6, *exam* examination, *max* maximal, *n a* not analyzed

### Latency duration of PPROM and neonatal respiratory parameters (primary outcomes)

First, correlation analysis between duration of PPROM and respiratory parameters as well as between gestational age at diagnosis of PPROM and respiratory parameters was performed using univariable analysis. We found significant correlations between duration of PPROM and the following parameters: surfactant application (*p* < 0.001; *r* = 0.397) and respiratory distress syndrome (*p* = 0.021; medians 204 and 98 h for children with and without respiratory distress syndrome). The other respiratory parameters (bronchopulmonary dysplasia (definition 36 weeks postmenstrual age), therapy with continuous positive airway pressure, therapy with diuretics, therapy with steroids) as well as neonatal parameters (birth weight, length at birth, head circumference at birth, year of birth, maximal C-reactive protein of infants in the first 72 postnatal hours, maximal Interleukin-6 of infants in the first 72 postnatal hours, clinical chorioamnionitis, histological chorioamnionitis) showed no correlation with PPROM duration (each *p*-value > 0.05). The respiratory parameters bronchopulmonary dysplasia (definition 36 weeks postmenstrual age), therapy with continuous positive airway pressure, therapy with diuretics, therapy with steroids, and the additional neonatal parameters (maximal CRP of infants in the first 72 postnatal hours, maximal Interleukin-6 of infants in the first 72 postnatal hours) showed no significant correlation with gestational age at diagnosis of PPROM (each *p* > 0.05).

As the numerous clinical parameters show multicollinearity between each other, we performed a multiple regression analysis using the “selection = stepwise” option for each outcome to consider this fact. Table 5 illustrates the results. Again, respiratory distress syndrome is significantly associated with gestational age at PPROM (*p* < 0.001), and surfactant application is significantly associated with PPROM duration and birth weight (*p* = 0.014 and *p* = 0.001). These results are confirmed by Table 3 demonstrating the comparison of different clinical parameters related to PPROM duration < 7 days and ≥ 7 days.

**Table 5:** Multiple regression analysis in order to investigate the influence of several parameters (gestational age at birth, birth weight, gestational age at PPROM and PPROM duration, clinical and histological chorioamnionitis) on different binary outcomes using multiple logistic regression analysis with “selection = stepwise” method

Influencing factor	Resp distress syndrome	Surfactant application	Broncho-pulmonary dysplasia	Therapy with continuous positive airway pressure	Therapy diuretics	Therapy steroids
Gestational age at birth	–	–	OR 1.453 *p* = 0.009	OR 1.503 *p* = 0.056	OR 1.369 *p* = 0.021	OR 0.1382 *p* = 0.028
Gestational age at PPROM	OR 0.671 *p* < 0.001	–	–	–	–	–
Birth weight	–	OR 0.182 *p* = 0.001	–	–	–	–
PPROM duration [weeks]	–	OR 1.294 *p* = 0.014	–	–	–	–
histological chorioamnionitis	–	–	–	–	–	–
Clinical chorioamnionitis	–	–	–	–	–	–
AUC	0.830	0.788	0.779	0.813	0.758	0.755

*Resp distress syndrome* respiratory distress syndrome, *OR* odds ratio, *AUC* area under the curve

For all outcomes in Table 5 (except for surfactant application), only one parameter has been chosen for the final models. Bronchopulmonary dysplasia, therapy with continuous positive airway pressure, diuretics, and steroids therapy revealed to be associated with gestational age at birth (each *p* < 0.05). Nevertheless, the AUC values ranging between 0.755 and 0.830 indicate a rather good statistical modelling.

### Latency duration of PPROM and bronchopulmonary dysplasia

Using the bronchopulmonary dysplasia definition with respiratory support at 36 weeks postmenstrual age (data available in 78 infants; Table 2), the duration of PPROM in the group with bronchopulmonary dysplasia (9 infants) was 1.8 ± 1.8 weeks (mean ± SD; median 0.9 weeks; range 0.4–6.0 weeks). In the group without bronchopulmonary dysplasia (69 infants), the PPROM duration was 2.0 ± 2.7 weeks (mean ± SD; median 1.0 weeks; range 0.3–16.1 weeks) (*p* = 0.737). Additionally, the occurrence of bronchopulmonary dysplasia or the bronchopulmonary dysplasia therapy (therapy with diuretics or steroids) was not significantly correlated with PPROM duration or gestational age at PPROM using regression analysis (Table 5). Furthermore, in the bronchopulmonary dysplasia group, PPROM was diagnosed at 25.7 ± 3.1 weeks of gestational age (mean ± SD; median 25.7 weeks; range 21.6–30.7 weeks) and in the group without bronchopulmonary dysplasia at 28.1 ± 3.9 weeks of gestational age (mean ± SD; median 28.4 weeks; range 14.4–34.9 weeks). This difference failed to be significant (*p* = 0.058). The rate of bronchopulmonary dysplasia in infants born after PPROM duration < 7 days in comparison to infants born after PPROM ≥ 7 days was not different (*p* = 1.000; Table 3).

### Latency duration of PPROM and neurodevelopmental parameter (secondary outcomes)

The neurological parameters (grade of intraventricular hemorrhage, MDI score (Bayley test), PDI score (Bayley test)) showed no correlation with PPROM duration nor with gestational age at diagnosis of PPROM (each *p*-value > 0.05; univariable analysis).

Periventricular leukomalacia was not observed in the study population. Testing of neurodevelopmental outcome of the included infants at a corrected age of 24 months using BSID II was available in 32 of the 84 included infants. This low number reflects the loss of follow-up or refusal of participation. In Germany, there is no organized national mandatory follow-up program or registry for long-term outcomes. Furthermore, neonatal care is highly decentralized; hence, follow-up visits are not necessarily performed at the center where the preterm infant was cared for. Our center is localized in a congested area with high urban mobility. Many patients grow up in families with low social status leading to fewer consultations at follow-up examinations. In these 32 tested infants, BSID II was performed at a corrected age of 25.4 ± 3.1 months (mean ± SD; median 25.5 months; range 16.0–31.0 months). The 32 tested infants revealed an MDI score of 95.4 ± 18.0 (mean ± SD; median 98.5; range 45.0–122.0). In 25 of the 32 tested infants, a PDI score was determined 86.6 ± 16.0 (mean ± SD; median 88.0; range 45.0–111.0). Additionally, infants born after PPROM duration < 7 days showed no significantly different neurodevelopmental outcome at a corrected age of 24 months from infants born after PPROM ≥ 7 days when determining MDI and PDI scores (Table 3). Interestingly, the scores of the group with PPROM ≥ 7 days were slightly higher without reaching significance.

## Discussion

Our study revealed that latency duration of PPROM as well as PPROM ≥ 7 days when compared with PPROM < 7 days are not associated with severe adverse respiratory or neurological outcome in pregnancies with careful monitoring. The infants with PPROM displayed solely an increased risk for pronounced respiratory distress syndrome and the respiratory distress syndrome can be treated very successful with surfactant preparations. Rapid begin of therapy with antibiotics after diagnosis of PPROM, observation of the women in the hospital, and prompt initiation of birth at the beginning of chorioamnionitis are important parts of a strategy to prolong gestation and to reduce morbidity in mother and fetus [3, 9].

We found no association between latency duration of PPROM and chorioamnionitis. This was confirmed by other investigators: Xie et al. found no significant correlation between latency duration and histological chorioamnionitis [4]. Latency duration was not associated with neonatal infection in our study population, and this observation also was confirmed by another study [14]. Additionally, Aziz et al. concluded that chorioamnionitis and neonatal sepsis were not associated with increased duration of latency [15].

In contrast to the results of this study, Pharande et al. reported about a higher incidence of chronic lung disease/mortality in infants with PPROM prior to 24 weeks’ gestation, but a lower incidence of chronic lung disease/mortality in infants with PPROM at or after 24 weeks in comparison to infants without PPROM [16]. Furthermore, Melamed et al. observed a higher rate of neonatal adverse outcomes in pregnancies with PPROM in comparison to pregnancies without PPROM, and neonatal adverse outcome was more prevalent when latency duration of PPROM was > 7 days [17]. Hanke et al. confirmed that PPROM per se is not associated with adverse outcome in very low birth weight infants with a gestational age of < 32 weeks; only a moderately increased risk for bronchopulmonary dysplasia (OR 1.90, 95% CI 1.29–2.79, *p* = 0.001) was observed in infants born between 27 and 32 weeks of gestation [18]. Development of bronchopulmonary dysplasia could be induced by PPROM with concomitant oligo-/anhydramnios [19]. The appearance of oligo-/anhydramnios might explain the higher need of surfactant according to higher grades of respiratory distress syndrome in infants with longer latency duration of PPROM. Severe pulmonary hypoplasia and persistent pulmonary hypertension are known complications after PPROM with anhydramnios [18, 20]. Interestingly, Thomas et al. described a short-term beneficial effect of histological, but not clinical chorioamnionitis on respiratory distress syndrome. This maturational effect is followed by a susceptibility of the lung for further postnatal injury increasing the risk for development of bronchopulmonary dysplasia [21].

A further study analyzed the vaginal bacteria relating to PPROM and showed that women with PPROM had a mixed, abnormal vaginal microbiota [22]. The microbiome profile at PPROM showed no correlation with gestational age at PPROM, latency duration, presence of chorioamnionitis, or neonatal outcome. Microbiome was remarkably variable during latency period of PPROM [22]. Chorioamnionitis is often induced by ascending genital microbes including genital mycoplasmas (*Ureaplasma urealyticum* and *Mycoplasma hominis*), *Gardnerella vaginalis* and *Bacteroides*, *Group B streptococcus*, and gram-negative bacteria such as *Escherichia coli* which are part of the vaginal or enteric flora [3]. This is in accordance with detection of *E. coli* in infants with early-onset sepsis and in the corresponding vaginal colonization of their mothers (Table 4). It is a well-established knowledge that infections with *E. coli* and *Klebsiella pneumoniae* are severe and often affected the brain resulting in disability. Additionally, sepsis with *E. coli* and *Klebsiella pneumoniae* often leads to acute renal failure and affected intestinal function. Early and hard treatment is essential to protect the organs from organ failure.

Neuroinflammatory injury not only destroys cerebral tissue, but may also change brain development [23]. Our study revealed no effect of PPROM duration on neurodevelopmental outcome at a corrected age of 24 months, although our follow-up rate was low. This was confirmed by another study concluding that for a given gestational age at birth, prolonged latency duration after PPROM does not worsen neonatal prognosis [24]. A further study of the University Hospitals Bonn and Essen, Germany, showed that PPROM ≥ 7 days did not influence neurodevelopmental outcome at a corrected age of 24 months [25]. In contrast to these results, Patkai et al. observed in a small cohort of very immature preterm infants with PPROM < 25 weeks a higher rate of delayed acquisitions (64.3% versus 15.8%), of behavioral disorders (57.1% versus 15.8%) and of lower language performance in two year old toddlers in comparison to children without PPROM (PPROM duration (mean ± SD) 16.6 ± 16.0 days). It must be mentioned that the number of infants with chorioamnionitis was greater in the group with PPROM in comparison to the group without PPROM [26]. A further study revealed that latency duration after PPROM of ≥ 3 weeks was an independent risk factor for MDI and PDI scores of < 70 [27]. Therefore, additional studies are needed to describe neurodevelopmental outcome after prolonged PPROM.

The presented study has some limitations: The study is not prospective, and the study population is small. Furthermore, the range of PPROM duration is wide. The results have to be confirmed by greater study groups and by other hospitals with similar obstetric-neonatological treatment. One additional limitation is that not every placenta was examined and that the histological reports about placental examination only delivered the information whether histological chorioamnionitis is present or not. Therefore, we are not able to report about severity of chorioamnionitis. It is important to recognize that an isolated maternal fever does not automatically correspond to chorioamnionitis. The term chorioamnionitis has been used excessively in clinical practice implying the presence of infectious and inflammatory conditions affecting the mother, fetus, and newborn [28]. As we recruit patients of the years 2005–2014, a time using this clinical practice, this concept was used for this retrospective study. Some years ago, the triple I concept was introduced in order to achieve better fetal, maternal, and neonatal care indicating intrauterine inflammation or infection or both while restricting the term chorioamnionitis to a pathologic diagnosis [28]. It was not senseful to use new criteria for a retrospectively analyzed collective because clinical decisions in this former time were not performed with Triple I theory. It is well-established that follow-up examinations of preterm infants are very important, but it is not always possible to realize this in all preterm infants due to loss of follow-up or refusal of participation. The results about neurodevelopmental outcome included only data of a part of the study population, and the results due to MDI and PDI have to be interpreted carefully. Additionally, there is a wide range of MDI and PDI scores in every group. It may be possible that a significant difference between different groups may be detected in a larger study population.

In conclusion, latency duration of PPROM is not associated with severe adverse neonatal outcome in expectantly and carefully managed pregnancies, but respiratory distress syndrome is associated with PPROM. The observed effect of pronounced respiratory distress syndrome can be effectively treated with surfactant preparations and is not followed by increased rate of bronchopulmonary dysplasia.

## Supplementary information

Below is the link to the electronic supplementary material.

Supplementary file1 (DOCX 35 kb)

## Abbreviations

CRP: C-reactive protein
MDI: Mental development index
PDI: Psychomotor developmental index
PMA: Postmenstrual age
PPROM: Preterm premature rupture of membranes
SD: Standard deviation
