# Supplementary material for: Latency duration of preterm premature rupture of membranes and neonatal outcome: a retrospective single-center experience
Source: Eur J Pediatr. 2021 Oct 4;181(2):801–11. doi: 10.1007/s00431-021-04245-2 (PMC8821059; doi:10.1007/s00431-021-04245-2)
Supplement: Supplementary file 1 — Supplementary file1 (DOCX 35 kb) [file 431_2021_4245_MOESM1_ESM.docx]

**Original research article for European Journal of Pediatrics**

**Title: Latency duration of preterm premature rupture of membranes and neonatal outcome: A retrospective single center analysis**

**Short running title:** Duration of PPROM and neonatal outcome

Hanna Müller^1,2^, Ann-Christin Stähling^2,3^, Nora Bruns^2^, Christel Weiss^4^, Maria Ai^5^, Angela Köninger^6^, Ursula Felderhoff-Müser^2^

^1^ Neonatology and Pediatric Intensive Care, Department of Pediatrics, University of Marburg, Baldingerstraße, 35043 Marburg, Germany

^2^ Department of Pediatrics I, Neonatology, Pediatric Intensive Care, Pediatric Neurology, University Hospital Essen, University Duisburg-Essen, Hufelandstr. 55, 45147 Essen, Germany

^3^ Clinic for Urology and Pediatric Urology, Marienhospital Marl, KKRN GmbH, Hervester Str.57, 45768 Marl, Germany.

^4^ Department of Medical Statistics and Biomathematics, University Hospital Mannheim, Theodor-Kutzer-Ufer 1-3, 68167 Mannheim, Germany.

^5^ Department of Pediatrics, University Hospital of Erlangen, University of Erlangen-Nürnberg, Loschgestr. 15, 91054 Erlangen, Germany

^6^ Department of Gynecology and Obstetrics, University Hospital Essen, University Duisburg-Essen, Hufelandstr. 55, 45147 Essen, Germany

**Full names, academic degrees and e-mail addresses of the authors:**

Prof. Dr. med. Hanna Müller, Hanna.Mueller@med.uni-marburg.de

Dr. med. Ann-Christin Stähling, acstaehling@gmail.com

Dr. med. Nora Bruns, Nora.Bruns@uk-essen.de

Prof. Dr. sc. hum. Christel Weiss, christel.weiss@medma.uni-heidelberg.de

Maria Ai, Maria.Ai@uk-erlangen.de

Prof. Dr. med. Angela Köninger, Angela.Koeninger@uk-essen.de

Prof. Dr. med. Ursula Felderhoff-Müser, Ursula.Felderhoff@uk-essen.de

**Corresponding author:**

Prof. Dr. Hanna Müller, Neonatology and Pediatric Intensive Care, Department of Pediatrics, University of Marburg, Baldingerstraße, 35043 Marburg, Germany

Phone: +49 - 6421 – 58-66973, Fax: +49 – 6421- 58-65917

Email address: Hanna.Mueller@med.uni-marburg.de

**SUPPLEMENTAL DATA**

**Supplemental Table 1** Association of clinical/histological chorioamnionitis with different clinical parameters

| **Histological chorioamnionitis** | | | |
| --- | --- | --- | --- |
|  | with histological  chorioamnionitis  (n = 13) | without histological  chorioamnionitis  (n = 45) | p value |
| PPROM duration in weeks (median, range) | 0.9 (0.3 – 13.6) | 1.1 (0.3 – 16.1) | 0.332 |
| Respiratory distress syndrome (n; %) | 10/13 (77%) | 31/42 (74%) | 1.000 |
| Max. grade of respiratory distress syndrome  (median, range) | 1 (0 – 4) | 2 (0 – 4) | 0.691 |
| Surfactant application (n; %) | 6/13 (46%) | 21/45 (47%) | 0.974 |
| numbers of surfactant applications  (median, range) | 0 (0 – 5) | 0 (0 – 4) | 0.518 |
| Grade of intraventricular hemorrhage  (median, range) | 0 (0 – 3) | 0 (0 – 0) | 0.580 |
| Therapy with continuous positive airway pressure (n; %) | 1/12 (8%) | 2/41 (5%) | 0.545 |
| Therapy with diuretics (n; %) | 2/11 (18%) | 3/44 (7%) | 0.259 |
| Therapy with steroids (n; %) | 0 | 3/42 (7%) | 1.000 |
| bronchopulmonary dysplasia (definition: 36 weeks PMA) (n; %) | 2/11 (18%) | 4/42 (10%) | 0.592 |
| bronchopulmonary dysplasia (definition: day 28 of life) (n; %) | 3/9 (33%) | 5/38 (13%) | 0.167 |
| MDI (mean ± standard deviation; range) | 89.8 ± 17.8 (66 – 115) | 97.9 ± 11.3 (78 – 111) | 0.224 |
| PDI (mean ± standard deviation; range) | 84.2 ± 16.1 (64 – 103) | 91.6 ± 12.6 (69 – 111) | 0.344 |
| **Clinical chorioamnionitis** | | | |
|  | with clinical  chorioamnionitis  (n = 41) | without clinical  chorioamnionitis  (n = 43) | p value |
| PPROM duration in weeks (median, range) | 1.0 (0.3 – 13.9) | 1.0 (0.3 – 16.1) | 0.275 |
| Respiratory distress syndrome (n; %) | 33/40 (82.5%) | 23/40 (57.5%) | 0.015 |
| Max. grade of respiratory distress syndrome  (median, range) | 1.5 (0 – 4) | 1 (0 – 4) | 0.049 |
| Surfactant application (n; %) ^b^ | 21/41 (51%) | 14/43 (33%) | 0.829 |
| numbers of surfactant applications  (median, range) | 1 (0 – 4) | 0 (0 – 5) | 0.303 |
| Grade of intraventricular hemorrhage  (median, range) | 0 (0 – 3) | 0 (0 – 2) | 0.912 |
| Therapy with continuous positive airway pressure (n; %) | 4/37 (11%) | 0 | 0.049 |
| therapy with diuretics (n; %) | 4/39 (10%) | 4/41 (10%) | 1.000 |
| therapy with steroids (n; %) | 3/39 (8%) | 4/39 (10%) | 1.000 |
| bronchopulmonary dysplasia (definition: 36 weeks PMA) (n; %) | 6/37 (16%) | 3/41 (7%) | 0.295 |
| bronchopulmonary dysplasia (definition: day 28 of life) (n; %) | 8/31 (26%) | 5/40 (12.5%) | 0.151 |
| MDI (mean ± standard deviation; range) | 91.8 ± 20.0 (45 – 115) | 98.9 ± 15.7 (54 – 112) | 0.271 |
| PDI (mean ± standard deviation; range) | 84.3 ± 16.8 (45 – 103) | 88.8 ± 15.6 (56 – 111) | 0.500 |
| **Histological OR clinical chorioamnionitis** | | | |
|  | with  chorioamnionitis  (n = 42) | without chorioamnionitis  (n = 25) | p value |
| PPROM duration in weeks (median, range) | 1.0 (0.3 – 13.9) | 1.2 (0.3 – 16.1) | 0.761 |
| Respiratory distress syndrome (n; %) | 34/41 (83%) | 12/23 (52%) | 0.009 |
| Max. grade of respiratory distress syndrome  (median, range) | 2 (0 – 4) | 1 (0 – 4) | 0.043 |
| Surfactant application (n; %) | 22/42 (52%) | 8/25 (32%) | 0.105 |
| numbers of surfactant applications  (median, range) | 1 (0 – 5) | 0 (0 – 3) | 0.202 |
| Grade of intraventricular hemorrhage  (median, range) | 0 (0 – 3) | 0 (0 – 1) | 0.684 |
| Therapy with continuous positive airway pressure (n; %) | 4/37 (11%) | 0 | 0.147 |
| therapy with diuretics (n; %) | 4/39 (10%) | 1/24 (4%) | 0.641 |
| therapy with steroids (n; %) | 3/39 (8%) | 1/22 (5%) | 1.000 |
| bronchopulmonary dysplasia (definition: 36 weeks PMA) (n; %) | 6/37 (16%) | 1/24 (4%) | 0.229 |
| bronchopulmonary dysplasia (definition: day 28 of life) (n; %) | 8/31 (26%) | 1/24 (4%) | 0.062 |
| MDI (mean ± standard deviation; range) | 91.8 ± 20.0 (45 – 115) | 100.0 ± 8.5 (85 – 110) | 0.167 |
| PDI (mean ± standard deviation; range) | 84.3 ± 16.8 (45 – 103) | 90.7 ± 15.7 (69 – 111) | 0.452 |

*MDI* mental development index, *PDI* psychomotor developmental index, *PMA* postmenstrual age

Statistical tests: Fisher's test; Cochran Armitage trend test; Chi2 test; t test
